# Overexpression of GPR41 attenuated glucose production in propionate-induced bovine hepatocytes

**DOI:** 10.3389/fvets.2022.981640

**Published:** 2022-09-02

**Authors:** Miao Lin, Maocheng Jiang, Tianyu Yang, Guoqi Zhao, Kang Zhan

**Affiliations:** ^1^Institute of Animal Culture Collection and Application, College of Animal Science and Technology, Yangzhou University, Yangzhou, China; ^2^Institutes of Agricultural Science and Technology Development, Yangzhou University, Yangzhou, China; ^3^Joint International Research Laboratory of Agriculture and Agri-Product Safety, The Ministry of Education of China, Yangzhou University, Yangzhou, China

**Keywords:** propionate, GPR41, hepatocytes, liver, glucose

## Abstract

Bovine liver mainly utilizes the propionate as a gluconeogenic substrate to synthesize the glucose. However, the mechanism underlying the regulatory effects of propionate on the glucose production in bovine hepatocytes remains less known. Previous studies have demonstrated G protein-coupled receptor 41 (GPR41) as receptors for propionate. We hypothesized that propionate may regulate the glucose production by GPR41 in bovine hepatocytes. Therefore, the aim of the study was to investigate the regulatory effects of propionate and GPR41 on glucose production in bovine hepatocytes. Hepatocytes with GPR41 overexpression were incubated in the presence of either 0 or 3 mM propionate for 24 h. These results showed that the expression of phosphoenolpyruvate carboxykinase 2 (PCK2) and pyruvate carboxylase (PC) genes involved in gluconeogenesis was enhanced (*P* < 0.01) with propionate treatment. Remarkably, the addition of propionate promotes the glucose production in bovine hepatocytes. Expression of GPR41 was increased by the addition of propionate in bovine hepatocytes overexpressed GPR41 by overexpression plasmid AAV1 compared with the absence of propionate. Interestingly, expression of PCK2 was markedly attenuated in GPR41 overexpressed-hepatocytes with propionate. Importantly, overexpression of GPR41 attenuated glucose output in propionate-induced bovine hepatocytes. These findings revealed that GPR41 negatively regulates glucose production by downregulating the expression of PCK2 in propionate-induced bovine hepatocytes.

## Introduction

Propionate is the most important gluconeogenic substrate for ruminants because it can provide more than 60 % of the carbon source for gluconeogenesis (1). In addition, the bovine liver also uses the amino acid, lactate, and pyruvate as gluconeogenic substrates to synthesize the glucose (2) to synthesize the glucose. However, the mechanism underlying the regulatory effects of propionate on the glucose production in bovine hepatocytes remains less known.

The hepatic gluconeogenesis process is regulated by several key factors such as pyruvate carboxylase (PC), glucose-6-phosphatase (G6PC), and phosphoenolpyruvate carboxykinase (PCK), which are the rate-limiting enzymes of the gluconeogenic pathway, and their activities reflect the extent of gluconeogenesis in the organism (3). The PC is responsible for the conversion of pyruvate in mitochondria to oxaloacetate (4). The cytosolic PCK1 is responsible for the conversion of oxaloacetate to phosphoenolpyruvate, which is a key part of cytoplasmic gluconeogenesis. However, oxaloacetate directly cannot pass through the mitochondrial membrane into the cytosol. Therefore, mitochondrial PCK2 plays a key role in the conversion of oxaloacetate to phosphoenolpyruvate in mitochondria (5). The phosphoenolpyruvate produces glucose-6-phosphate by a series of reactions, and then glucose-6-phosphatase catalyzes the hydrolysis of glucose-6-phosphate to generate glucose. The previous study has demonstrated that propionate can enhance the PC, PCK1, and PCK2 mRNA levels of genes involved in the gluconeogenic pathway in bovine hepatocytes (6). However, the mechanism of the regulatory effects of propionate on the genes involved in a gluconeogenic pathway in bovine hepatocytes that are unknown.

Some transmembrane genes play a major role in cell physiology and metabolism (7). G protein-coupled receptors 41 (GPR41) act as receptors for propionate (8). It is well-known that Gα subunits have been classified into four families, Gα (s), Gα (i/o), Gα (q), and Gα (9) protein (10). The GPR41 is coupled to the Gα (i/o) family of G proteins, and which inhibits the intracellular levels of second messengers cAMP. The G protein-coupled receptors OLFR734 can increase the intracellular levels of cAMP and expression of PCK1 and G6PC, eventually promoting glucose production in mouse hepatocytes (11). We hypothesized that GPR41 may negatively regulate the glucose production in propionate-induced bovine hepatocytes. Therefore, the aim of this study was to determine the regulatory effects of propionate and GPR41 on gluconeogenic genes and glucose production in bovine hepatocytes.

## Methods

This study was carried out in accordance with the principles of Yangzhou University, the Institutional Animal Care and Use Committee [SYXK (Su) IACUC 2012-0029]. Isolation of primary hepatocytes from three mid-lactating Holstein cows was performed as described previously (12). The cows were fed a TMR to meet 100 % of NRC requirements and milked on the third dairy at 8:00, 14:00, and 21:00, respectively. All cows were free of clinical signs of disease before the isolation of primary hepatocytes. To determine the effects of propionate on the expression of genes involved in the gluconeogenic pathway and glucose production, hepatocytes were cultured in 6-well plates at a density of 2 × 10^5^/well and grown at 37°C, 5 % CO_2_, and 95 % humid conditions. After incubation for 12 h, cells were incubated in the presence of either 0 (as a control group) or 3 mM propionate for 24 h (9). Total RNA was isolated from the cultured cells using a TRIzol kit (Tiangen, Beijing, China). Reverse transcription (RT) was performed using an RT Kit (Takara, Beijing, China). Before the qRT-PCR for samples, the amplification efficiencies of all primers were determined by using a standard dilution series (Table 1). For glucose output, the medium was then replaced with 1 ml of glucose-free DMEM and supplemented with 10 mM lactate and 1 mM sodium pyruvate. After incubation for another 2 h, the glucose level in the medium was determined by the kit (Applygen, E1011, Beijing, China). Results were normalized to protein content.

**Table 1:** Primers for real-time PCR analysis.

**Gene**	**Primer sequence, 5' to 3'**	**Accession number**	**Source**
*PCK2*	F: 5 TGACTGGGCAAGGGGAGCCG 3 R: 5 GGGGCCACCCCAAAGAAGCC 3	NM_001205594.1	Zhang et al. (6)
*PC*	F: 5 CCACGAGTTCTCCAACACCT 3 R: 5 TTCTCCTCCAGCTCCTCGTA 3	NM_177946.4	Zhang et al. (6)
*G6PC*	F: 5 TGATGGACCAAGAAAGATCCAGG 3 R: 5TATGGATTGACCTCACTGGCCCTCTT 3	NM_001076124.2	Zhang et al. (6)
*GPR41*	F: 5 AACCTCACCCTCTCGGATCT 3 R: 5 GCCGAGTCTTGTACCAAAGC 3	NM_001145233.1	Wang et al. (13)
*GAPDH*	F: 5 GGGTCATCATCTCTGCACCT 3 R: 5 GGTCATAAGTCCCTCCACGA 3	NM_001034034	Zhan et al. (9)

F, forward; R, reverse.

The bovine GPR41 CDS gene was obtained using bovine cDNA as a template by PCR amplification. The pAAV expression plasmid vector (Clontech, Beijing, China) was linearized by EcoRI and BamHI (NEB, Beijing, China) restriction enzyme digestion. The pAAV-GPR41 vector was constructed according to the In-Fusion cloning reaction (Clontech) and extracted by the Free Endotoxin Maxi Plasmid Kit (Tiangen, Beijing, China). The pAAV-GPR41 vector, pHelper, and pRC1 (AAV1) (Addgene, Beijing, China) or pAAV-GFP vector (Takara, Dalian, China; as control) were transfected into 293T cells to package AAV1 serotypes using polyethyleneimine Max (Ployscience, Shanghai, China), respectively. AAV1 viruses were obtained by a 0.22 μm filtration membrane. The CT values of the respective viral gradients were obtained by qRT-PCR, and the standard curve of the AAV virus was established. The final AAV1 vg was calculated by the average of AAV1 viruses vg from 10 and 200 fold dilution (Table 1). Bovine hepatocytes were designed with MOI = 10^6^ values and infected with AAV1 for 3 days, and next hepatocytes with overexpressed GPR41 were treated in the presence of either 0 or 3 mM propionate for 24 h.

### Statistical analysis

The statistical analysis was tested by the independent sample *t*-test, using SPSS 19.0 software (SPSS Inc.; Chicago, IL, USA). *P* < 0.05 was considered significant, and < 0.01 were highly significant.

## Results

**Figure 1:**
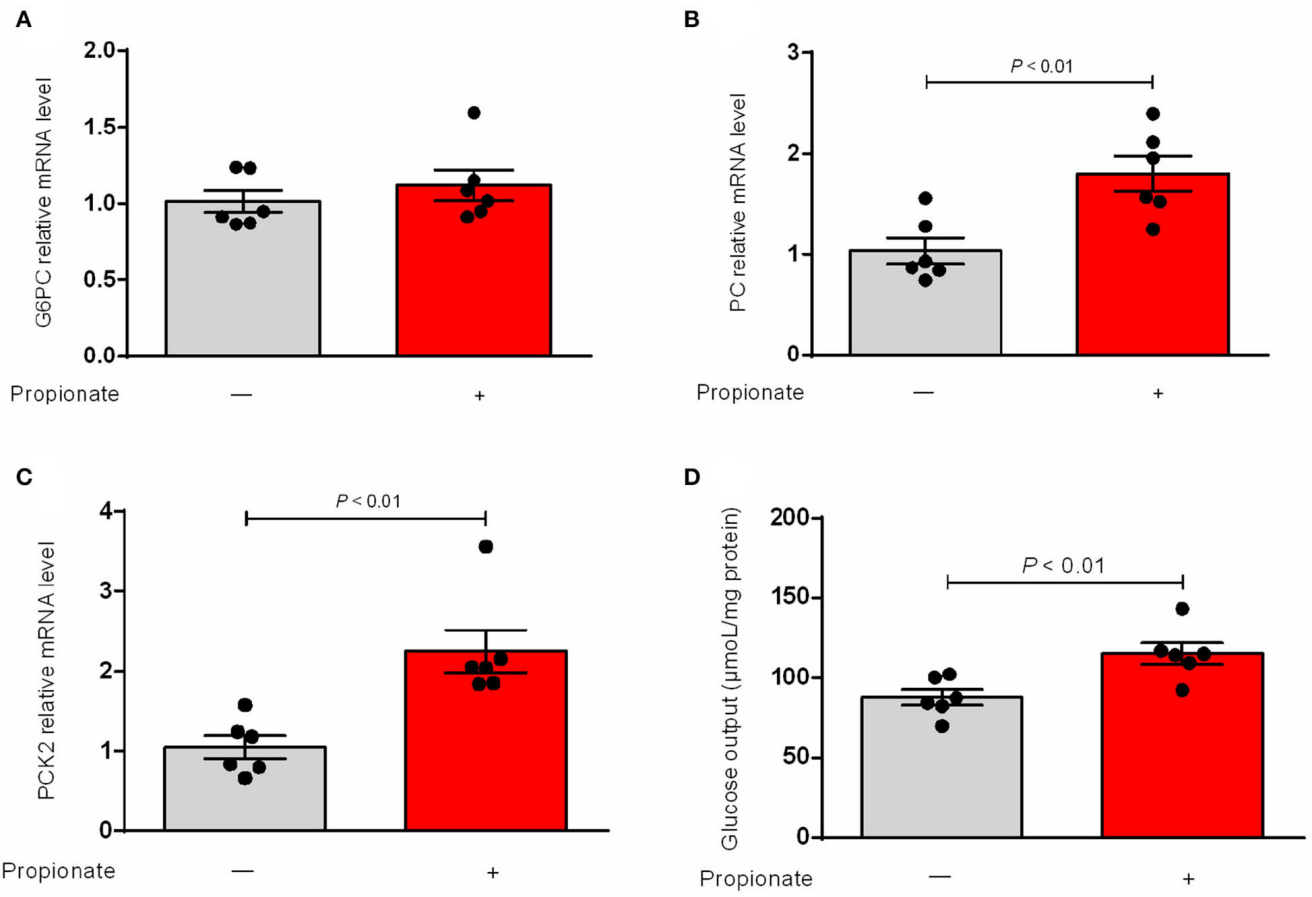
Effect of propionate on mRNA relative level of genes involved in gluconeogenic and glucose output in bovine hepatocytes. qRT PCR analysis of G6PC **(A)**, PC **(B)**, and PCK2 **(C)** expression levels (*n* = 6). GAPDH was used as an internal reference gene. **(D)** Propionate on the content of glucose output in bovine hepatocytes (*n* = 6). The relative expression of target genes was normalized to that of GAPDH. *P* < 0.05 were considered significant and <0.01 were highly significant.

**Figure 2:**
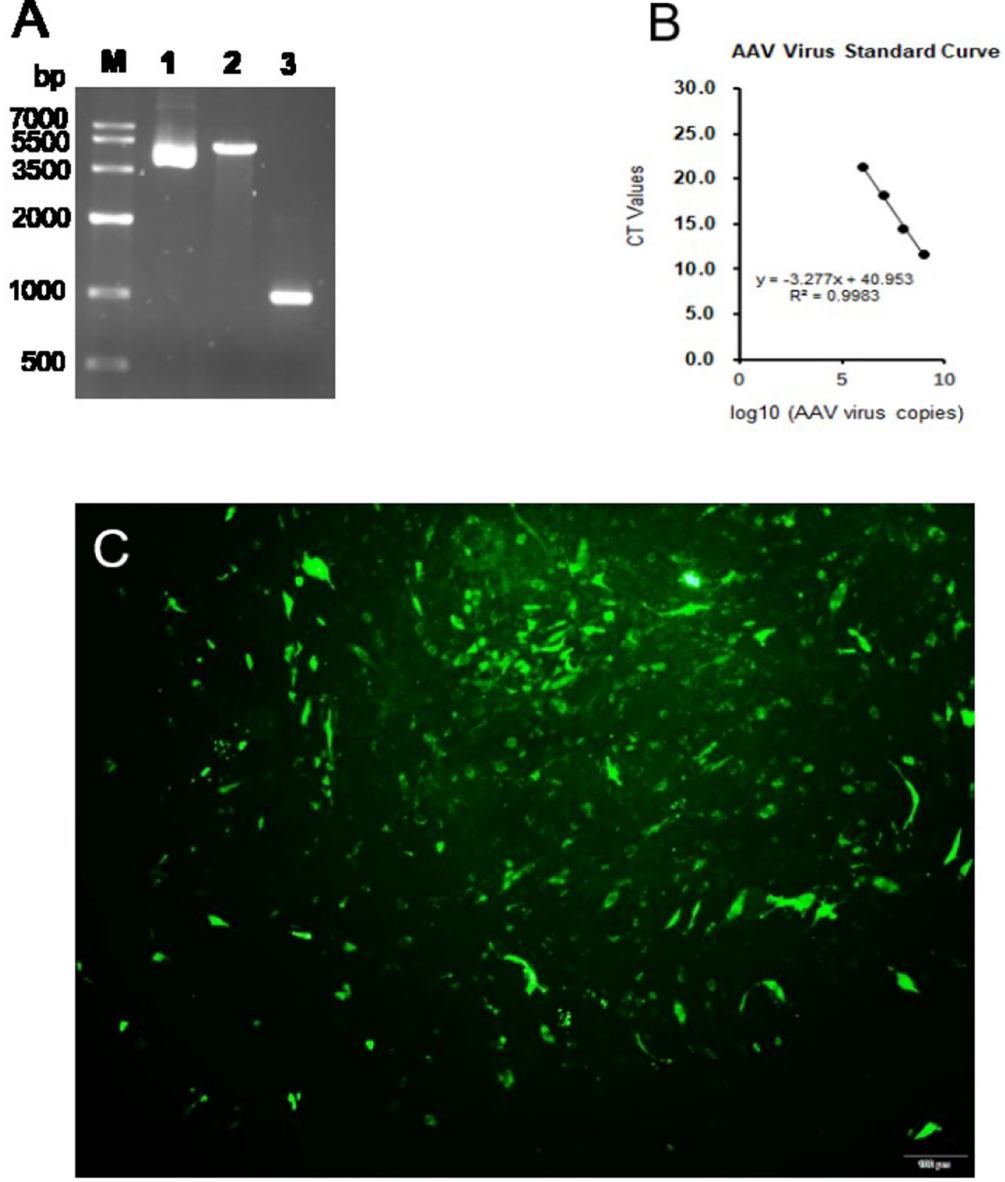
pAAV-GPR41 vector construction and virus. **(A)** Lane 1, pAAV expression plasmid vector. Lane 2, pAAV vector linearized by EcoRI and BamHI. Lane 3, GPR41 amplification by pAAV-GPR41 as template. **(B)** Standard curve of AAV virus. **(C)** Bovine hepatocytes infected by the pAAV-GFP virus.

**Figure 3:**
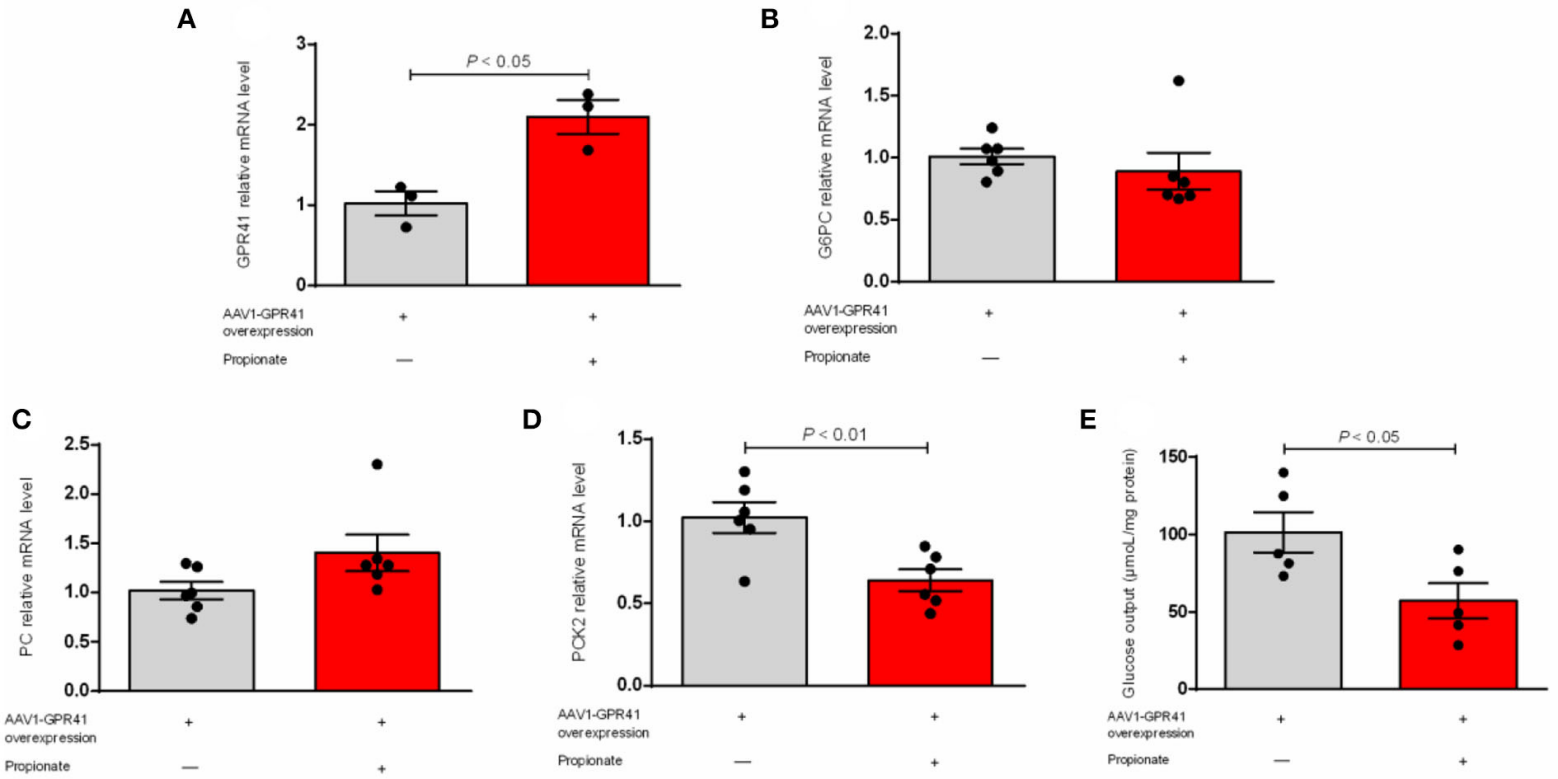
Effect of GPR41 overexpression on the mRNA relative level of genes involved in gluconeogenic and glucose output in propionate-induced bovine hepatocytes. qRT-PCR analysis of GPR41 **(A)**, G6PC **(B)**, PC **(C)**, and PCK2 **(D)** expression levels (*n* = 6). **(E)** GPR41 overexpression on the content of glucose output in propionate induced bovine hepatocytes (*n* = 6). The relative expression of target genes was normalized to that of GAPDH. *P* < 0.05 were considered significant, and <0.01 were highly significant.

Our result showed that the expression of G6PC was not changed (Figure 1A). The hepatic mRNA abundance of PC and PCK2 was enhanced (*P* < 0.01) by the incubation of propionate compared with control (Figures 1B,C). The addition of propionate promotes (*P* < 0.01) the glucose output compared with control (Figure 1D). The pAAV-GPR41 vector and standard curve of the AAV virus were successfully constructed (Figures 2A,B). The transduction efficiency of AAV1 exhibited a significant effect for 3 days at MOI = 10^6^ in hepatocytes (Figure 2C). The expression of GPR41 was elevated (*P* < 0.05) in propionate-induced bovine hepatocytes with GPR41 overexpression compared with the lack of propionate (Figure 3A). The overexpression of GPR41 did not affect expression of G6PC and PC in propionate-induced bovine hepatocytes relatively to lack of propionate (Figures 3B,C). However, overexpression of GPR41 attenuated (*P* < 0.05) expression of PCK2 and glucose production in propionate-induced bovine hepatocytes compared with the lack of propionate (Figures 3D,E).

## Discussion

In the present study, propionate enhanced the gene expression of PCK2 and PC and glucose production. However, expression of PCK2 was attenuated in GPR41 overexpressed-hepatocytes with propionate. Remarkably, overexpression of GPR41 decreased glucose output in propionate-induced bovine hepatocytes. These findings demonstrated that overexpression of GPR41 attenuated glucose production in propionate-induced bovine hepatocytes by downregulating the expression of PCK2 in propionate-induced bovine hepatocytes.

We confirmed that propionate enhanced the mRNA abundance of PCK2 and PC, but the effect of propionate on G6PC was not found. The present results are consistent with the previous studies that showed propionate induces mRNA expression of rate-limiting enzyme genes involved in the gluconeogenic pathway in bovine hepatocytes (14). However, the study did not investigate the effect of propionate on the glucose output in bovine hepatocytes. Our results showed that propionate induces the glucose output. To date, little is known about the molecular mechanism underlying the regulatory effects of propionate on the glucose production in bovine hepatocytes. Previous studies identified GPR41 as a receptor for propionate (8). The interaction of bovine GPR41 with propionate triggers the inhibition of the cAMP level (15). The G protein-coupled receptors OLFR734 can enhance the intracellular levels of cAMP, eventually promoting the glucose production in mouse hepatocytes (11). We thus hypothesized that GPR41 may regulate the glucose production by changing the expression of certain genes related to the gluconeogenic pathways in propionate-induced bovine hepatocytes.

To further explore the effect of GPR41 on the regulation of genes related to a gluconeogenic pathway and glucose production in propionate-induced bovine hepatocytes, we overexpressed GPR41 using AAV1. Propionate can induce the mRNA expression of GPR41 compared with the lack of propionate. However, overexpression of GPR41 reduced the expression of PCK2 in propionate-induced bovine hepatocytes compared with the lack of propionate. The mitochondrial PCK2 plays a key role in the conversion of oxaloacetate to phosphoenolpyruvate in mitochondria (5). The PCK2 approximately accounts for 1 % and 5 % of the total phosphoenolpyruvate carboxykinase activity in mouse and rat livers, respectively (9). However, PCK1 and PCK2 exhibited an equal activity in ruminants (16). The propionate as a gluconeogenic substrate to synthesize the glucose depends on the PCK2 activity in goat hepatocytes (15). Phosphoenolpyruvate produced by PCK2, as a gluconeogenic precursor, can pass directly through the mitochondrial membrane into the cytoplasm for gluconeogenesis, indicating that PCK2 plays a key role in the gluconeogenesis pathway in ruminants. Importantly, overexpression of GPR41 reduced glucose output in propionate-induced bovine hepatocytes compared with the lack of propionate. The PCK2 is the rate-limiting enzyme of the gluconeogenic pathway, which can regulate glucose output in hepatocytes. An attenuation in an expression of PCK2 by overexpression of GPR41 consistently decreased the glucose production in propionate-induced bovine hepatocytes. The GPR41 couples to the Gα (i/o) family of G proteins in mammalian cells, and activation of the Gi pathway suppress forskolin-induced cAMP production. However, cells overexpressed with an unrelated GPCR, the endothelin-B receptor, did not induce the cAMP production (17). A previous study also demonstrated that bovine GPR41 was overexpressed to trigger the interaction of bovine GPR41 with propionate, resulting in the inhibition of the cAMP signaling (13). OLFR734, G protein-coupled receptors, promotes the hepatic cAMP levels to induce the glucose production in mouse hepatocytes (11). In the present study, propionate enhanced the expression of GPR41 to promote the interaction of GPR41 and Gα (i/o) protein, leading to a decrease in glucose production. In this study, overexpression of GPR41 attenuated the expression of PCK2 and glucose production in propionate-induced bovine hepatocytes. These findings revealed that GPR41 negatively regulates glucose production by downregulating the expression of PCK2 in propionate-induced bovine hepatocytes.

In conclusion, GPR41 as a receptor for propionate potentially provides a molecular link between GPR41 and glucose production, and GPR41 negatively regulates glucose production by downregulating the expression of PCK2 in propionate-induced bovine hepatocytes.

## Data availability statement

The original contributions presented in the study are included in the article/supplementary material, further inquiries can be directed to the corresponding author.

## Ethics statement

This experiment was performed under protocols approved by the Animal Care and Use Committee of Yangzhou University, Yangzhou, China, and Ethic Code is SYXK (Su) IACUC 2012-0029. The authors confirm that they have followed EU standards for the protection of animals used for scientific purposes.

## Author contributions

ML and MJ performed the experiment, including chemical analysis, and statistical analysis. ML and TY wrote the manuscript. GZ and KZ conducted the study, manuscript writing, and manuscript revision. All authors have read and approved the final version of this manuscript.

## Funding

This study was supported by the Research Project of Natural Science Foundation of Jiangsu Province (BK20190898), the National Natural Science Foundation of China (No. 32002200), and supported by the earmarked fund for CARS (CARS-36).

## Conflict of interest

The authors declare that the research was conducted in the absence of any commercial or financial relationships that could be construed as a potential conflict of interest.

## Publisher's note

All claims expressed in this article are solely those of the authors and do not necessarily represent those of their affiliated organizations, or those of the publisher, the editors and the reviewers. Any product that may be evaluated in this article, or claim that may be made by its manufacturer, is not guaranteed or endorsed by the publisher.
